# Mowing Did Not Alleviate the Negative Effect of Nitrogen Addition on the Arbuscular Mycorrhizal Fungal Community in a Temperate Meadow Grassland

**DOI:** 10.3389/fpls.2022.917645

**Published:** 2022-06-09

**Authors:** Siqi Qin, Guojiao Yang, Yang Zhang, Meixia Song, Lu Sun, Yangzhe Cui, Jibin Dong, Ning Wang, Xiao Liu, Peiming Zheng, Renqing Wang

**Affiliations:** ^1^School of Life Sciences, Institute of Ecology and Biodiversity, Shandong University, Jinan, China; ^2^Shandong Provincial Engineering and Technology Research Center for Vegetation Ecology, Shandong University, Jinan, China; ^3^Qingdao Forest Ecology Research Station of National Forestry and Grassland Administration, Shandong University, Jinan, China; ^4^College of Ecology and Environment, Hainan University, Haikou, China

**Keywords:** arbuscular mycorrhizal fungi, nitrogen addition, diversity, community composition, environmental filtering

## Abstract

As nitrogen deposition intensifies under global climate change, understanding the responses of arbuscular mycorrhizal (AM) fungi to nitrogen deposition and the associated mechanisms are critical for terrestrial ecosystems. In this study, the effects of nitrogen addition and mowing on AM fungal communities in soil and mixed roots were investigated in an Inner Mongolia grassland. The results showed that nitrogen addition reduced the α-diversity of AM fungi in soil rather than that of root. Besides, nitrogen addition altered the composition of AM fungal community in soil. Soil pH and inorganic nitrogen content were the main causes of changes in AM fungal communities affected by nitrogen addition. Mowing and the interaction of nitrogen addition and mowing had no significant effect on AM fungal community diversity. In contrast, while mowing may reduce the negative effects of nitrogen addition on the richness and diversity of plants by alleviating light limitation, it could not do so with the negative effects on AM fungal communities. Furthermore, AM fungal communities clustered phylogenetically in all treatments in both soil and roots, indicating that environmental filtering was the main driving force for AM fungal community assembly. Our results highlight the different responses of AM fungi in the soil and roots of a grassland ecosystem to nitrogen addition and mowing. The study will improve our understanding of the effects of nitrogen deposition on the function of ecosystem.

## Introduction

Human activities have caused increasing atmospheric nitrogen deposition that has seriously affected the biodiversity and functioning of terrestrial ecosystems (Smith et al., 1999; Yu et al., 2019). For plants, nitrogen deposition can affect both plant community composition and diversity (Wei et al., 2013; Zhang et al., 2014) due to soil acidification, aluminum toxicity (Wei et al., 2013), light limitation (Borer et al., 2014), and increased nitrogen availability (Liu et al., 2020). Meanwhile, lower pH and increased nitrogen availability following nitrogen deposition can lead to alteration in the soil microbial community structure and reductions of species diversity (Wang et al., 2018; Liu et al., 2020). Moreover, grasslands are mowed to harvest hay, one of the most important ways of managing grassland ecosystems. Previous studies have shown that mowing can mitigate the negative effects of nitrogen deposition on plant diversity by removing litter and alleviating light limitation (Luo et al., 2019; Yang et al., 2019). However, information on the impact of mowing on belowground microbial communities under the background of nitrogen deposition in grassland ecosystems is limited.

Arbuscular mycorrhizal (AM) fungi can form mutualistic associations with most land plants, thereby providing mineral nutrients to their plant partners in exchange for the photosynthetic products used to maintain growth and function (Lu and Hedin, 2019; Tedersoo et al., 2020). In addition, AM fungi have important ecological functions in the succession, biodiversity, productivity, and material and energy cycling of terrestrial ecosystems (Vályi et al., 2016; Powell, 2018; Tedersoo et al., 2020). A recent meta-analysis showed that nitrogen deposition significantly decreased the AM fungal abundance (Han et al., 2020). However, some studies have shown that fertilization has no significant or positive effect on the AM fungal community (Zheng et al., 2014; Pan et al., 2020). The response of the AM fungal community to nitrogen deposition may be affected by different ecosystem types as well as the rates and duration of nitrogen deposition (Han et al., 2020; Ma et al., 2020). Nitrogen is limited resource in grassland ecosystems, and nitrogen deposition removes plants from nutrient limitation. So that plants are less dependent on AM (Olsson et al., 2010). AM obtain less photosynthetic products from host plants (Pearson and Jakobsen, 1993; Smith et al., 1999), thereby intensifying competition among AM fungi and changing their community composition and structure. Moreover, nitrogen deposition may alter plant community composition, and host plant may also actively select AM fungal taxa that can help the host plants obtain more benefits. (Veresoglou and Rillig, 2014). Researches of the effects of mowing on AM fungal community are also controversial. Mowing altered the community composition of AM fungi through soil properties (Sepp et al., 2018). In addition, there were different views that mowing has no significant effects on the species composition of AM fungi (Zubek et al., 2022) and the colonization of AM fungi in roots (Eom et al., 1999). However, it remains unclear whether mowing alters the effects of nitrogen deposition on AM communities in grassland ecosystems.

In general, deterministic processes are important in regulating ecological communities when environmental filtering and competitive exclusion contribute to community assembly, and stochastic processes are important when dispersal and chance shape community assembly (Vályi et al., 2016). However, nitrogen deposition may affect the relative importance of deterministic and stochastic processes. Nitrogen deposition and mowing can change soil environmental variables, and the assembly process of AM fungal community is influenced by soil pH, soil nutrients, and soil type (Johnson, 2010; Liu et al., 2012). Furthermore, spore dispersal is an important way for AM fungi to disperse (Janos et al., 1995). Spore density of AM fungi is altered after nitrogen deposition (Zheng et al., 2014), which affects the dispersal process of AM fungi. The phylogenetic structure of AM fungi has been used to clarify the dominant ecological processes that drive AM fungal community assembly (Webb et al., 2002). Environmental filtering, stochastic processes, and competitive exclusion may lead to phylogenetic clustering, random distributions, and over-dispersion of the AM fungal community, respectively (Webb et al., 2002). An eight-year fertilization experiment showed that the phylogenetic pattern of AM fungal communities shifted from clustering under no fertilization to a random distribution under low fertilization treatments and over-dispersion under high fertilization treatments (Liu et al., 2015). Chen et al. (2017) found that AM fungal communities were phylogenetically clustered according to nitrogen deposition, suggesting that environmental filtering was the main driver of AM fungal community assembly. At present, evidence is inconclusive concerning whether the ecological processes driving AM fungal community assembly under nitrogen deposition are consistent.

In this study, long-term (seven years) nitrogen addition and mowing experiments in the temperate grassland of Inner Mongolia were conducted. We investigated the composition of the plant community and used high-throughput sequencing to study how plant and AM fungal communities responded to nitrogen addition and mowing. We addressed three questions: (1) How do plant and AM fungal communities respond to nitrogen addition? We assumed that the diversity of the plant community and the AM fungal community would decrease with increasing nitrogen addition. (2) Will mowing change the responses of plant and AM fungal communities to nitrogen addition? We hypothesized that mowing would alleviate the negative effects of nitrogen addition on the composition and diversity of the plant and AM fungal communities. (3) What are the main ecological processes responsible for the AM fungal community assembly under nitrogen addition and mowing treatments? We hypothesized that environmental filtering was the primary driver of AM fungal community assembly.

## Materials and Methods

### Study Site and Experimental Design

The study site was located in a temperate meadow grassland in Inner Mongolia of northern China (50°10′N, 119°22′E). Mean annual temperature and precipitation at the site are −1.59°C and 336.5 mm (2000–2020), respectively. The study area has been fenced since 2013 to avoid disturbance by large animals. Vegetation is dominated by *Leymus chinensis*, *Stipa baicalensis*, *Cleistogenes squarrosa*, *Thermopsis lanceolate*, *Cymbaria daurica*, and *Carex duriuscula*.

A nitrogen addition experiment was initiated in 2014 and has been maintained since then. Nitrogen fertilization were added when grasslands turned green. The nitrogen fertilization was mixed with the roasted fine sand and uniformly spread into the corresponding experimental plots. A series of 10 m × 10 m plots were laid out and separated by 1 m walkways. We selected five nitrogen rates (0, 2, 5, 10, and 20 g m^−2^ yr.^−1^) of urea for the nitrogen addition experiment. Mowing treatments (non-mown and mown) were set up based on nitrogen addition, with a total of 10 treatments (nitrogen addition without mowing treatments: N0, N2, N5, N10, N20; Nitrogen addition with mowing treatments: MN0, MN2, MN5, MN10, MN20), each replicated five times. The mowing treatment was carried out at the end of August every year, and plants were mown with a height of about 10 cm remaining.

### Plant and Soil Sampling

Samples of plant species were collected in August by clipping all plants at the soil surface using a 1 m × 1 m quadrat randomly placed in each plot. After bringing the plant sample back to the laboratory, plants were dried (105°C for 1 h, and 65°C for 48 h) and weighed for dry mass. Soil samples were collected from the top 15 cm of the soil profile using a soil core sampler (6 cm internal diameter). Six soil cores were collected from each plot after the plant biomass harvest and mixed to give one composite sample. Thus, a total of 50 mixed stratified soil samples were collected. The soil samples were passed through a 2.0 mm sieve and homogenized. Plant roots were carefully picked out with tweezers. Roots and a portion of fresh soil were stored at −80°C for DNA extraction and − 20°C for inorganic N analysis, and the remaining soil was air-dried and stored at room temperature for determination of soil chemical properties.

### Soil Properties Analyses

Soil moisture was determined as the mass loss after drying the soil at 105°C for 24 h. The soil pH was measured with a soil-water ratio of 1:5 using a pH meter. The ammonium ( ${\mathrm{NH}}_{4}^{+}‐N$ ) and nitrate nitrogen ( ${\mathrm{NO}}_{3}^{-}‐N$ ) were leached with 2 mol L^−1^ KCl and determined by MgO-Devarda alloy distillation. The total nitrogen (TN) and phosphorus (TP) contents were digested with concentrated sulfuric acid, then determined by the Kjeldahl nitrogen and molybdenum antimony colorimetric methods, respectively. The total carbon content (TC) of the soil was measured using potassium dichromate heating. The available phosphorus content (AP) of the soil was leached with NaHCO_3_ and determined by the molybdenum antimony colorimetric method.

### DNA Extraction and Sequencing

Mycorrhizal fungal DNA was extracted from the soil and mixed root samples using a Fast DNA SPIN Kit (MP Biomedicals LIC, Santa, Ana, CA). The quality and quantity of the extracted DNA were determined by electrophoresis on a 2.0% agarose gel. All DNA samples were amplified by nested PCR. The first step was performed with the primer pair AML1F (5′-ATCAACTTTCGATGGTAGGATAGA-3′) and AML2R (5′-GAACCCAAACACTTTGGTTTCC-3′; Lee et al., 2008). The PCR mixture consisted of 4 μl of 5 × FastPfu Buffer, 2 μl of dNTPs mixture (each 2.5 mM), 0.8 μl of each primer, 0.4 μl of FastPfu polymerase, 0.2 μl of BSA, and 10 ng of template DNA combined with sterile deionized H_2_O to a total volume of 20 μl. The thermal cycling conditions were an initial denaturation at 95°C for 3 min, 32 cycles of denaturation at 95°C for 30 s, annealing at 55°C for 30 s, and extension at 72°C for 45 s followed by a final extension at 72°C for 10 min. The PCR products were further amplified in the second step with the primer pair AMV4-5NF (5′-AAGCTCGTAGTTGAATTTCG-3′) and AMDGR (5′-CCCAACTATCCCTATTAATCAT-3′; Sato et al., 2005). Except for the number of cycles being set at 30, the PCR conditions for the second PCR were similar to the first PCR.

PCR products were sent to Majorbio Bio-pharm Technology Co., Ltd., (Shanghai 201203, China) for Illumina MiSeq sequencing. The raw high-throughput sequencing data were first processed using the Quantitative Insights into Microbial Ecology (QIIME)^1^ toolkit. 2365781 valid readings were obtained after quality control filtering. We clustered our sequences into operational taxonomic units (OTUs) with the criterion of sequence identity ≥97% and used the representative sequence of each OTU to Blast against the MaarjAM database^2^ for taxonomic assignment (Öpik et al., 2010). After normalization, 924 OTUs were obtained, of which 573 OTUs were identified at the family level with known AM fungal families. The raw reads were submitted to the NCBI Sequence Read Archive (SRA) database under the accession numbers PRJNA790751.

### Statistical Analyses

In this study, the structural equation modeling (SEM) was performed using the AMOS software (IBM SPSS AMOS 24.0.0), other statistical analysis and graphing were performed in R (R version 4.0.5). Richness, Shannon-Wiener index (H), Simpson index (D), and Pielou index (E) were used to measure the α-diversity of the community.


$$H=-\overset{s}{\underset{i=1}{\sum }}p_{i}\log_{2}p_{i}$$

$$D=1-\overset{s}{\underset{i=1}{\sum }}p_{i}{}^{2}$$

$$E=\frac{H}{\log_{2}N}$$

Where *p_i_* is relative biomass of plant species *i* or OTU abundance of AM fungi *i*. *N* is plant species richness or OTU abundance of AM fungi. *H* is Shannon-Wiener index; *D* is Simpson index. *E* is Pielou index.

Two-way ANOVA was used to examine the effects of N addition, mowing and their interaction on soil properties, plant and AM fungal communities. The effects of different nitrogen addition rates on soil properties and plant and AM fungal α-diversity were tested using one-way ANOVA followed by Tukey HSD test for each mown or unmown treatment. The differences between mown and unmown treatments were tested by independent *t* tests. Before the ANOVA, we performed a normality test and a test for the homogeneity of variance on all data. For the data that did not satisfy the assumptions of normality of the distribution or the homogeneity of variance, the logarithmic, reciprocal, or square root transformations were performed, and the nonparametric Kruskal-Wallis test was used for the data that still did not satisfy the conditions after the transformation.

Principal component analysis (PCA) was conducted to reduce the number of variables for plant and AM fungal community composition using the R package ‘vegan’. The AM fungal community structure was visualized by non-metric multidimensional scaling (NMDS) ranking based on the Bray-Curtis distance matrices using the ‘metaMDS’ function of the R package ‘vegan’ (Faith et al., 1987). Permutational multivariate analysis of variance (PERMANOVA, 999 permutations) was used to test the effects of nitrogen addition and mowing on the AM fungal community structure by using the ‘adonis’ function of the R package ‘vegan’ (Warton et al., 2012).

Structural equation modeling was used to examine the causal pathways by which nitrogen addition and mowing affected the AM fungal community. Based on our knowledge of the effects of nitrogen deposition and mowing on the AM fungal community, we constructed *a priori* model (Supplementary Figure S1). The generalized least squares method was used to fit the data into the model. Chi square, root mean square error of approximation (RMSEA), and goodness of fit index (GFI) were used to assess model fit (Grace, 2006). We calculated NRI and NTI using the ‘ses.mpd’ and ‘ses.mntd’ functions (NRI and NTI were equivalent to −1 times output of ‘ses.mpd’ and ‘ses.mntd’) of the R package ‘picante’, respectively (Webb et al., 2008; Chen et al., 2017).

## Results

### Responses of Soil Parameters and Plant to Nitrogen Addition and Mowing

Nitrogen addition significantly reduced the soil pH from 6.86 to 5.72 (*F* = 23.65, *p* < 0.001; Table 1). TN, ${\mathrm{NH}}_{4}^{+}‐N$ , ${\mathrm{NO}}_{3}^{-}‐N$ , and inorganic nitrogen in the soil increased with the increase of nitrogen addition, but only the N10 or N20 treatments were significantly different from the controls (*F* = 5.356, *p* < 0.01; *F* = 6.956, *p* < 0.01; *F* = 3.588, *p* < 0.05; *F* = 7.947, *p* < 0.001; Table 1). Nitrogen addition had no significant effect on soil TC, TP, or AP. When nitrogen addition and mowing were carried out simultaneously, the response of soil parameters to nitrogen addition was consistent with that of only nitrogen addition (Table 1). Mowing did not significantly change soil properties (*p* > 0.05, results not shown).

**Table 1:** Soil properties tested by one-way ANOVA in N and MN treatments [results are means ± SE, *n* = 5, except $NH_{4}^{+}N$ , $NO_{3}^{-}N$ , Inorganic N of MN5 (*n* = 4)] and summary of results of ANOVA (*F*-values and significance levels).

	Soil moisture %	pH	Total C (mg/g)	Total N (mg/g)	Total P (mg/g)	Available P (mg/kg)	$NH_{4}^{+}N$ (mg/kg)	$NO_{3}^{-}N$ (mg/kg)	Inorganic N (mg/kg)
N0	15.81 ± 0.465	6.86 ± 0.102a	26.10 ± 1.533	2.58 ± 0.071b	0.43 ± 0.011	2.58 ± 0.429	7.87 ± 0.990b	6.44 ± 1.396b	14.31 ± 1.222c
N2	14.63 ± 0.565	6.57 ± 0.041b	23.22 ± 1.404	2.55 ± 0.148b	0.41 ± 0.011	2.96 ± 0.499	8.73 ± 1.522b	7.21 ± 1.289b	15.94 ± 2.350bc
N5	14.41 ± 0.905	6.46 ± 0.107b	22.26 ± 0.752	2.71 ± 0.084b	0.41 ± 0.012	2.47 ± 0.142	7.62 ± 1.018b	5.31 ± 1.151b	12.92 ± 1.676c
N10	14.78 ± 0.540	5.94 ± 0.058c	24.82 ± 2.295	2.82 ± 0.093b	0.41 ± 0.016	3.00 ± 0.265	19.10 ± 5.766a	11.02 ± 2.948ab	30.12 ± 8.624b
N20	15.20 ± 0.652	5.72 ± 0.140c	24.70 ± 2.787	3.21 ± 0.154a	0.42 ± 0.026	3.30 ± 0.490	39.93 ± 9.995a	16.79 ± 3.552a	56.71 ± 12.609a
Significance of									
N	0.737	23.65^***^	0.625	5.356^**^	0.313	0.76	6.956^**^	3.588^*^	7.947^***^
MN0	14.02 ± 0.757	6.90 ± 0.063a	24.96 ± 2.611	2.60 ± 0.052bc	0.43 ± 0.014	2.24 ± 0.347	7.85 ± 0.756 cd	5.97 ± 1.826bc	13.82 ± 2.262c
MN2	13.84 ± 0.536	6.80 ± 0.095a	22.72 ± 1.442	2.32 ± 0.112c	0.38 ± 0.015	2.28 ± 0.490	7.00 ± 0.507d	4.41 ± 0.424c	11.41 ± 0.800c
MN5	13.69 ± 0.527	6.26 ± 0.104b	24.98 ± 1.193	2.77 ± 0.086ab	0.41 ± 0.014	3.07 ± 0.427	10.79 ± 0.62bc	8.83 ± 1.848abc	19.62 ± 2.256bc
MN10	13.38 ± 0.837	6.05 ± 0.074b	24.00 ± 0.968	2.94 ± 0.164ab	0.45 ± 0.015	2.77 ± 0.233	17.70 ± 4.603ab	9.85 ± 1.324ab	27.55 ± 4.495b
MN20	12.54 ± 0.524	5.42 ± 0.109c	24.02 ± 1.628	3.06 ± 0.166a	0.40 ± 0.021	3.49 ± 0.352	45.38 ± 12.708a	12.11 ± 1.814a	57.49 ± 13.519a
Significance of									
MN	0.805	43.84^***^	0.336	5.596^**^	2.689	1.975	10.93^***^	4.22^*^	12.14^***^

N0, N2, N5, N10 and N20, nitrogen addition with 0, 2, 5, 10, and 20 g m^−2^ yr^−1^ respectively; MN0, MN2, MN5, MN10, and MN20, mowing and nitrogen addition with 0, 2, 5, 10 and 20 g m^−2^ yr^−1^, respectively. Different letters denote significant (*p* < 0.05) differences among treatments. ^*^p < 0.05, ^**^p < 0.01, ^***^p < 0.001. N, the effect of nitrogen addition; MN, the effect of mowing and nitrogen addition.

**Figure 1:**
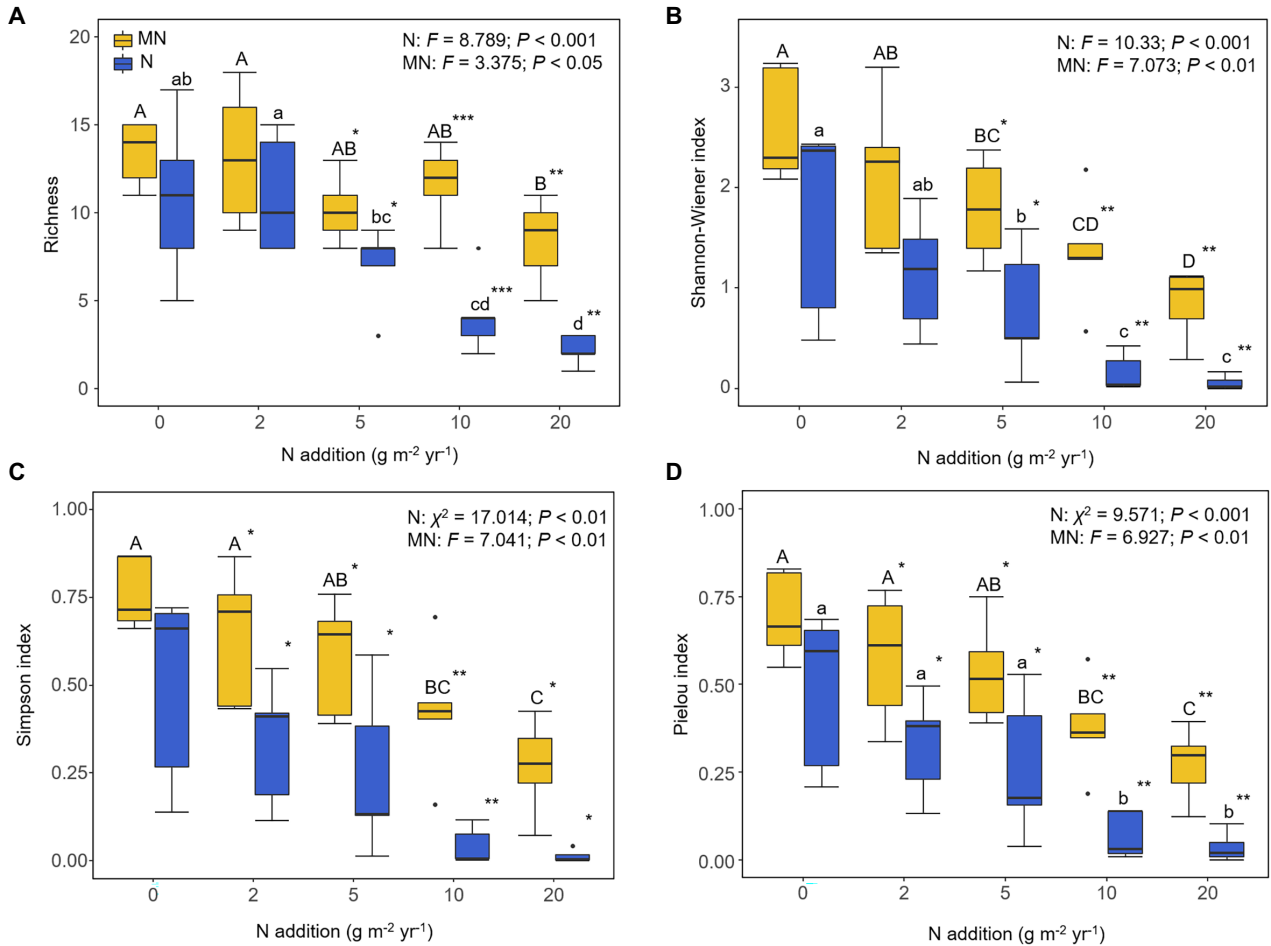
Effects of nitrogen addition (N) and mowing (M) on plant α-diversity: **(A)** plant richness, **(B)** plant Shannon-Wiener index, **(C)** plant Simpson index, and **(D)** plant Pielou index. One-way ANOVA was used to test the response of plant α-diversity in each nitrogen addition and mowing treatment. Different lower- and upper-case letters denote significant difference (*p* < 0.05) among nitrogen addition rates in the mown and unmown treatments. The symbol “*” at the right corner of lower- and upper-case letters indicates a significant difference between mown and unmown treatments at each nitrogen addition rate (*p < 0.05, **p < 0.01, ***p < 0.001). Treatments 0, 2, 5, 10 and 20 denote nitrogen addition with 0, 2, 5, 10 and 20 g m^−2^ yr.^−1^, respectively.

Plant richness and the Shannon-Wiener, Simpson, and Pielou indices were significantly reduced under the nitrogen addition (*p* < 0.001; Figure 1), where plant richness was reduced to about two species under N20. After mowing, the plant richness and Shannon-Wiener index were significantly improved at N5 and higher treatments compared with only nitrogen addition (*t*-test, *p* < 0.001; Figure 1). At the same time, the Simpson and Pielou indices were significantly increased under N2 and higher treatments (*t*-test, *p* < 0.001; Figure 1).

### Responses of AM Fungal Communities in Soil and Roots to Nitrogen Addition and Mowing

**Figure 2:**
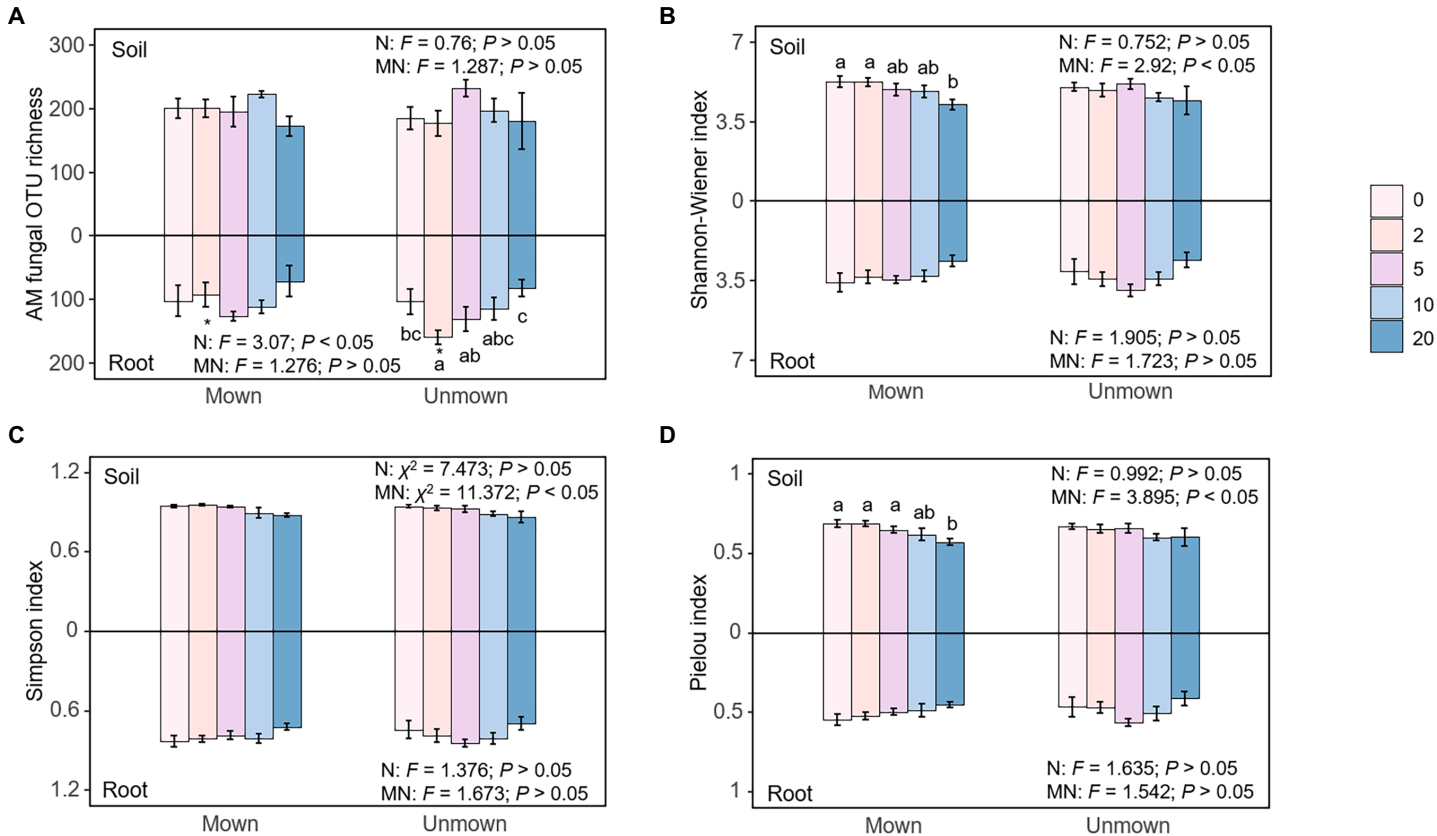
Effects of nitrogen addition (N) and mowing (M) on AM fungal α-diversity in soil (upper) and roots (lower): **(A)** AM fungal operational taxonomic unit (OTU) richness, **(B)** AM fungal Shannon-Wiener index, **(C)** AM fungal Simpson index, **(D)** AM fungal Pielou index. All data are expressed as mean ± SE (*n* = 5). One-way ANOVA, Tukey’s HSD, and *t* tests were used to test the effects of nitrogen addition and mowing on AM fungal α-diversity in the soil and roots in each nitrogen addition and mowing treatment. Different letters denote significant differences (*p* < 0.05) among nitrogen addition rates in the mown and unmown treatments at soil or root. The symbol “*” at the top or bottom of the column indicates a significant difference (*p* < 0.05) between mown and unmown at each nitrogen addition rate in soil or root. The numbers 0, 2, 5, 10, and 20 denote nitrogen addition with 0, 2, 5, 10, and 20 g m^−2^ yr.^−1^, respectively.

There was no significant difference in AM fungal operational taxonomic unit (OTU) richness in the soil under all treatments (Figure 2A). The Shannon-Wiener and Pielou indices of AM fungal communities in the soil under mowing conditions were significantly different only under MN20 compared with the control treatment, but Shannon-Wiener, Simpson, and Pielou indices generally decreased with the increase of nitrogen addition (Shannon-Wiener: *p* < 0.05, Simpson: *p* < 0.05, Pielou: *p* < 0.05; Figures 2B–D). Nitrogen addition only exerted an influence on the OTU richness of AM fungal community in roots (*p* < 0.05, Figure 2A), while no other significant difference was found in the Shannon-Wiener, Simpson, and Pielou indices of AM fungal communities in the roots in other treatments (*p* > 0.05, Figures 2B–D). Mowing did not change the richness or α-diversity indices of AM fungal communities in the soil or roots (*t*-test, Figure 2).

**Figure 3:**
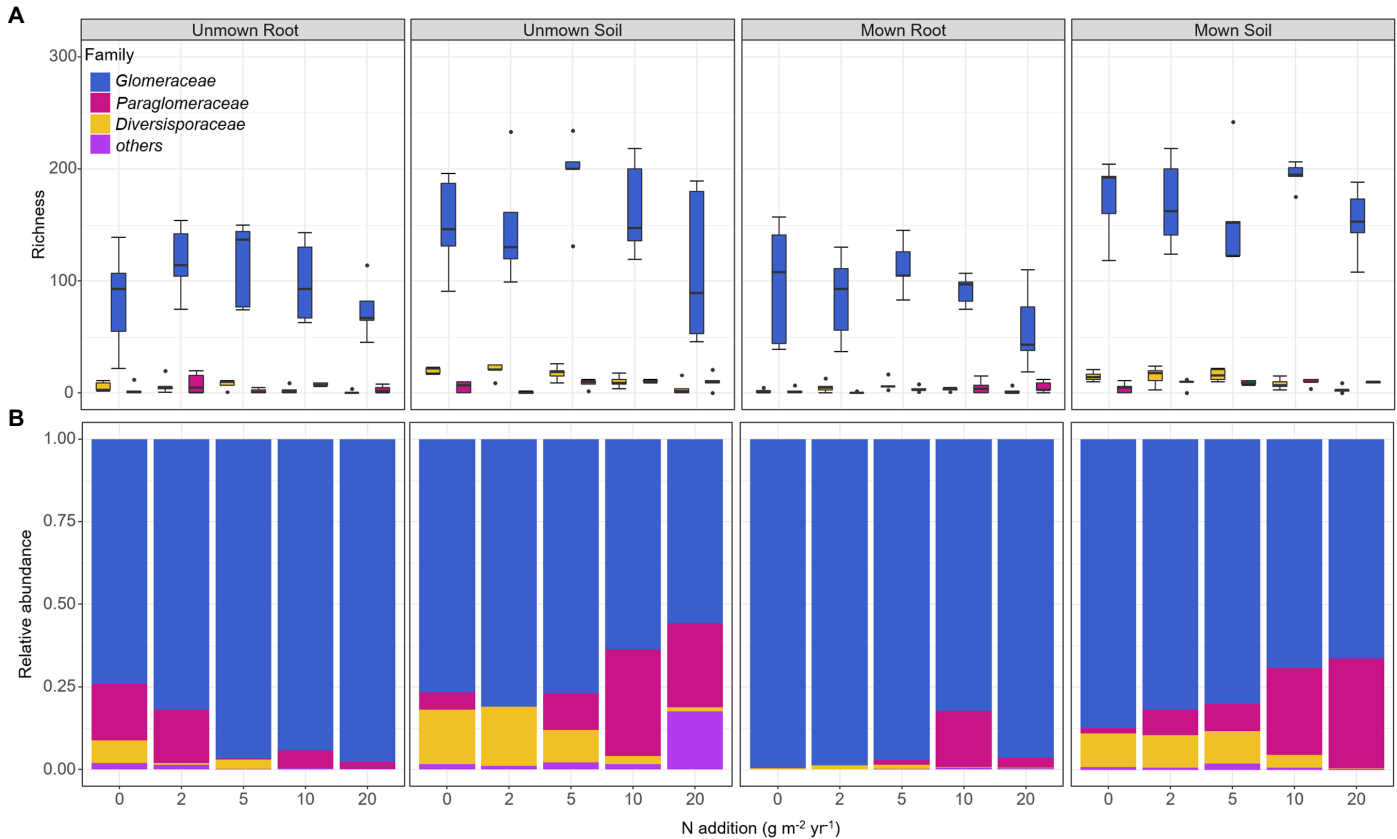
Effects of nitrogen addition and mowing on the richness and relative abundance of AM fungal dominant families. **(A)** Richness of AM fungal dominant families and **(B)** relative abundance of AM fungal dominant families. The mid-horizontal lines within boxplots represent the median. 0, 2, 5, 10, and 20 denote nitrogen addition with 0, 2, 5, 10, and 20 g m^−2^ yr.^−1,^ respectively.

**Figure 4:**
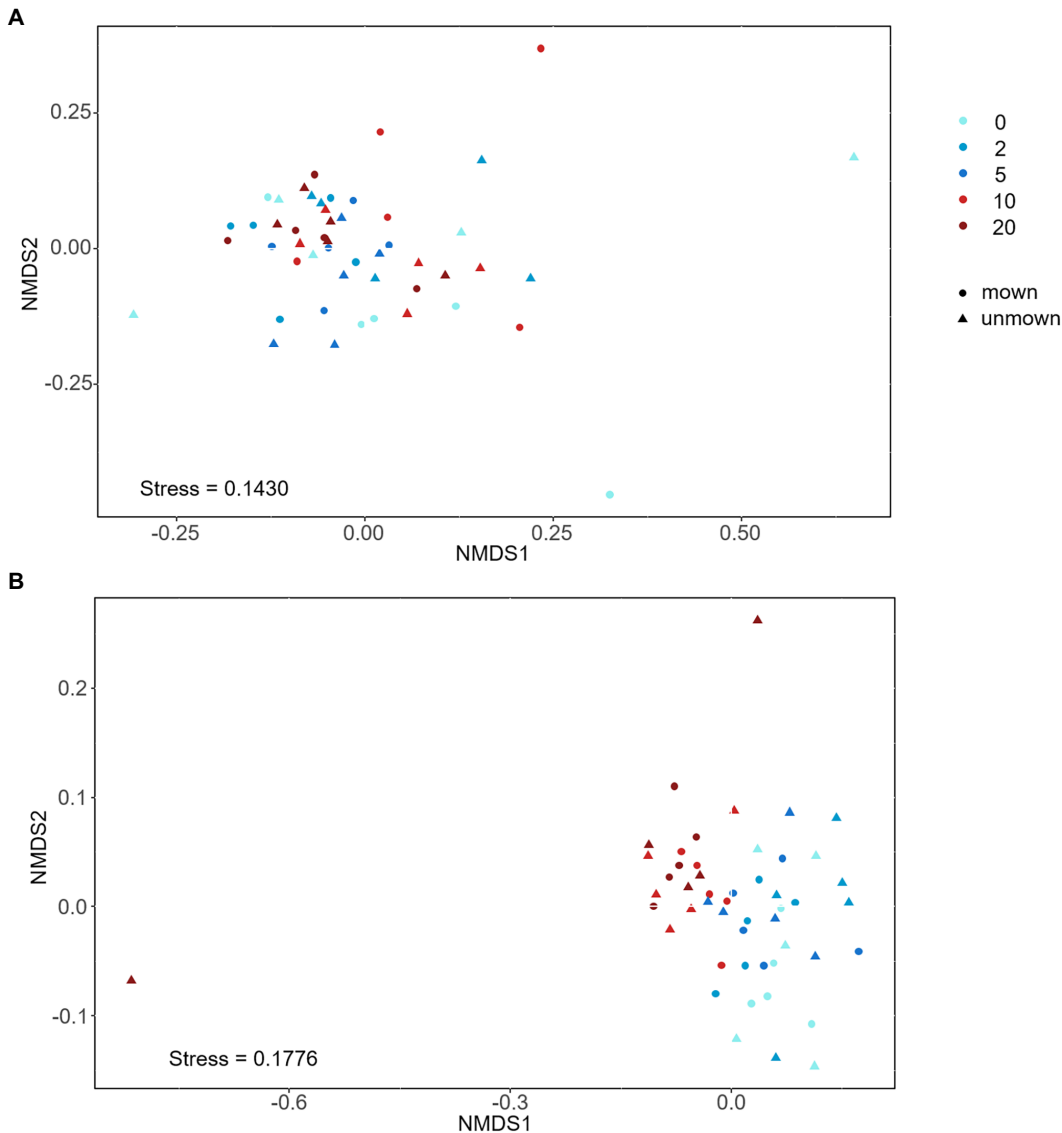
Nonmetric multidimensional scaling (NMDS) analysis of AM fungal community structures under nitrogen addition: **(A)** AM fungal community composition in roots, **(B)** AM fungal community composition in soil. 0, 2, 5, 10, and 20 denote nitrogen addition with 0, 2, 5, 10, and 20 g m^−2^ yr.^−1^, respectively.

The OTU richness of Glomeraceae was more diverse and had higher ratio reads than other families in all treatments (Figure 3A). Regardless of mown or unmown treatment, the relative abundances of Glomeraceae and Diversisporaceae families decreased with increasing nitrogen addition in the soil; the Paraglomeraceae family showed an opposite trend to the Glomeraceae and Diversisporaceae families (Figure 3B). However, Glomeraceae assumed a supermajority of the percentage in the roots (Figure 3B). Regardless of soil or roots, both AM fungal community composition and beta diversity were significantly affected by nitrogen addition (PERMANOVA, *R*^2^ = 22.18%, *p* = 0.001; *R*^2^ = 11.31%, *p* = 0.041; Table 2). There was no detectable effect of mowing on AM fungal community structure in the soil or roots (Table 2; Figure 4).

**Table 2:** Results from a permutational multivariate analysis of variance (PERMANOVA) testing the effects of nitrogen addition, mowing, and the interaction on Bray–Curtis dissimilarity of AM fungal community in soil and roots, respectively.

	Treatment	*R* ^2^	*P*
Root	*M*	1.69%	0.551
	*N*	11.31%	**0.041**
	M × N	9.25%	0.204
Soil	*M*	2.63%	0.072
	*N*	22.18%	**0.001**
	M × N	6.79%	0.461

*N*, the effect of nitrogen addition; *M*, the effect of mowing; N × M, the interaction of nitrogen addition with mowing. Bold means a significant difference (p < 0.05).

**Figure 5:**
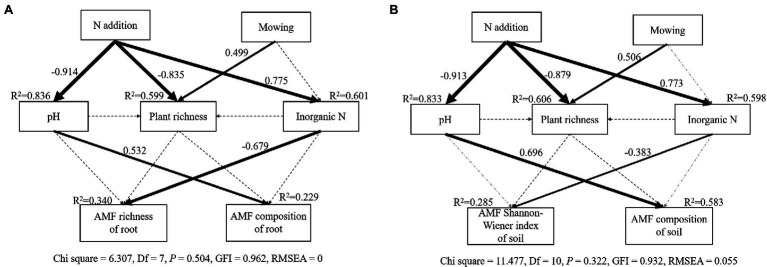
The structural equation modeling (SEM) analysis of the effects of nitrogen addition and mowing on AM fungal richness, community composition in roots **(A)** and AM fungal community Shannon-Wiener index, community composition in soil **(B)** *via* the pathways of nitrogen addition, mowing, soil pH, soil inorganic N, plant richness. Numbers adjacent to arrows are path coefficients, and width of the arrows is proportional to the strength of path coefficients. Black dashed arrows indicate non-significant relationships (*p* > 0.05). Gray dashed arrows indicate paths removed to improve model fits. Percentages close to endogenous variables indicate the variance explained by the model (*R*^2^).

The SEM model adequately fitted data describing the interaction pathways between plants, AM fungal communities, and soil parameters in response to nitrogen addition and mowing (Soil: Chi square = 11.477, Df = 10, *p* = 0.322, GFI = 0.932, RMSEA = 0.055; Root: Chi square = 6.307, Df = 7, *p* = 0.504, GFI = 0.962, RMSEA = 0; Figure 5). The final model explained 22.9, and 34% of the variation in AM fungal community composition and richness in roots (Figure 5A), and explained 58.3, and 28.5% of the variation in AM fungal community composition and Shannon-Wiener index in soil, respectively (Figure 5B). Regardless of in soil or roots, soil pH showed significant correlations with AM fungal composition, whereas inorganic N showed significant correlations with AM fungal richness of root or AM fungal Shannon-Wiener index of soil (Figure 5).

### Ecological Process of AM Fungal Community Assembly

**Figure 6:**
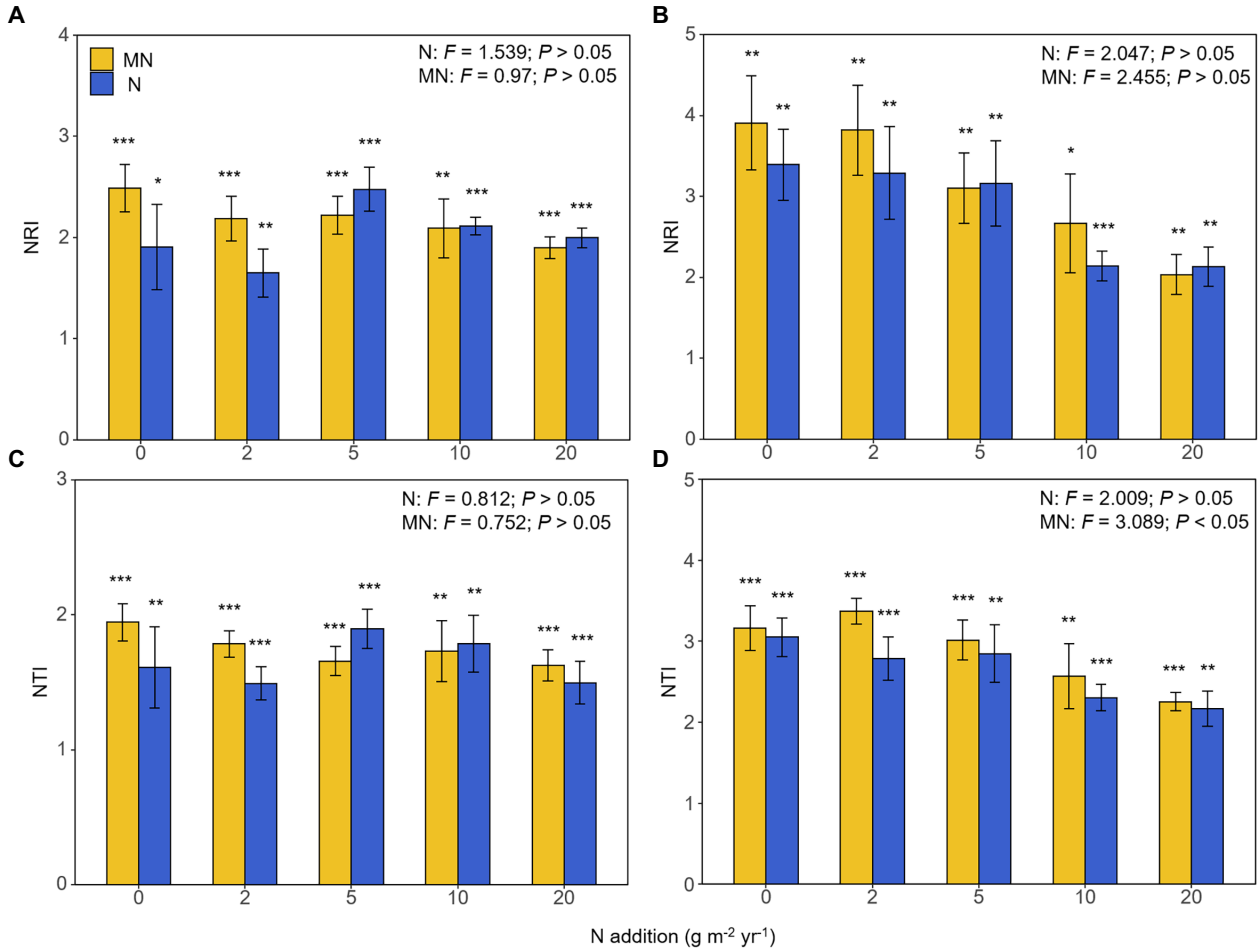
The responses of the nearest relative index (NRI) of the arbuscular mycorrhizal fungal community in roots **(A)** and soil **(B)** to nitrogen addition and mowing treatments, and nearest taxa index (NTI) of the arbuscular mycorrhizal fungal community in roots **(C)** and soil **(D)** to nitrogen addition and mowing treatments. N, the effect of nitrogen addition; MN, the effect of mowing and nitrogen addition. ^*^p < 0.05 of *t-*test, ^**^p < 0.01 of *t*-test, ^***^p<0.001 of *t*-test.

The NRI and NTI of the AM fungal community were all significantly greater than zero (Figure 6), indicating that AM fungal species were more closely related than expected by chance in all treatments. In other words, the phylogenetic structure of AM fungal communities was always clustered in all treatments. Except that the treatment of mowing under nitrogen addition had a significant effect on the NTI of the AM fungal community in soil (*p* < 0.05, Figure 6D), there was no significant difference in the NRI and NTI of AM fungal communities in other treatments (*p* > 0.05, Figure 6). However, the NRI and NTI of the AM fungal community had a downward trend across the nitrogen addition rates (but were always greater than zero), which only occurred in the soil (Figure 6).

## Discussion

### The Effects of Nitrogen Addition and Mowing on Plant Community

Consistent with previous research (Clark and Tilman, 2008; Tian et al., 2016), nitrogen addition reduced the diversity of plant community (Figure 1). We found that the soil pH decreased and the inorganic nitrogen content increased significantly after nitrogen addition (Table 1). In addition, resource competition and soil acidification were considered to be the main causes of species loss (Stevens et al., 2004; Farrer and Suding, 2016; Greaver et al., 2016). On the one hand, nitrogen addition led to competition for limiting resources among different species or functional groups, including competition for light from aboveground resources (Grime, 1973; Newman, 1973) and competition for nutrients from belowground resources (Supplementary Figure S2A; Wei et al., 2013). On the other hand, soil acidification increased concentrations of harmful Al^3+^, Mn^2+^, and Fe^3+^ (Maskell et al., 2010), thereby reducing plant diversity (Supplementary Figure S2B).

Previous studies have shown that mowing can reduce the negative impact of nitrogen on plant species diversity by removing the nitrogen accumulated in the soil (Storkey et al., 2015; Tilman and Isbell, 2015). However, mowing did not change inorganic N concentrations in the present study (Table 1) but did mitigate the negative effects of nitrogen addition on plant diversity (Figure 1). Nitrogen addition promoted the rapid growth of some nitrogen-loving plants, which caused shading to other plants in the community, resulting in the loss of these shaded plant species from the community due to light limitation (Grime, 1973; Newman, 1973). Mowing may have slowed the light competition by changing plant height. Unfortunately, we did not quantify the availability of light in this experiment; this prevented us from directly assessing the role of light restriction in explaining the mechanisms generating plant diversity.

### The Effects of Nitrogen Addition and Mowing on the AM Fungal Community

As expected, N addition significantly caused negative effects on AM fungal diversity in soil (Figure 2), supporting our first hypothesis. On the one hand, nitrogen addition can increase the inorganic nitrogen content and lower the soil pH, toxic ions released from soil acidification and increased ammonium toxicity both affected AM fungal community composition (Da Silva et al., 2014; De Beenhouwer et al., 2015). On the other hand, increased soil nitrogen availability makes host plant less dependent on AM (Olsson et al., 2010). The benefits that plants provide to AM were reduced, intensifying competition among AM fungi. Moreover, nitrogen addition may indirectly affect AM fungal communities through changing plant community (Chen et al., 2014). However, we did not find a significant relationship between plant richness and AM fungal community in the SEM (Figure 5). This may be due to the correlation between the plant and AM fungi being strong after short-term nitrogen addition, but the correlation weakens after long-term treatment (Li et al., 2015). The study site had been adding nitrogen for 7 years at the time of sampling. Long-term nitrogen addition may have weakened the relationship between plants and AM fungal communities. Therefore, it is necessary to carry out long-term monitoring of the changes of plant and AM fungal communities. Contrary to our second hypothesis, mowing did not alleviate the negative impact of nitrogen addition on AM fungal diversity (Figures 2, 3), possibly because nitrogen addition reduced AM fungal diversity by modulating soil inorganic nitrogen, but mowing did not change soil properties, especially soil pH and inorganic nitrogen content (Table 1), in our experiments.

We found significant differences between soil and root AM fungal community composition (Figure 3). Some studies have also shown that Paraglomeraceae and Glomeraceae are dominant in soil, but Glomeraceae are dominant in roots (Powell et al., 2009; Varela-Cervero et al., 2015). AM fungi occupy different ecological niches in time and space. In addition, the evolutionary dynamics that drive the formation of AM fungal communities in soil and root systems are different, resulting in different AM fungal community compositions in roots and soil (Pringle and Bever, 2002; Liu et al., 2012). The present results indicated that nitrogen addition affected the AM fungal community composition in soil but not in roots (Figure 3B), consistent with the study of Lü et al. (2020). The effect of nitrogen addition on the soil AM fungal community composition can be attributed to changes in soil nutrients (Zheng et al., 2014). As such, AM fungi in soil may be more sensitive to changes in soil nutrients, while AM fungi in roots are more closely associated with the roots of the host plant. The decrease in the richness and relative abundance of Glomeraceae in the soil with nitrogen addition (Figure 3) suggested that some members of the family have greater carbon requirements, nitrogen addition can promote down-regulation by the host or competitive exclusion among members of the family (Kiers et al., 2011; Duenas et al., 2020).

### Ecological Process of AM Fungal Community Assembly

Consistent with the third hypothesis, environmental filtering was a major process of AM fungal community assembly under mowing and nitrogen deposition. The role of environmental filtration has been demonstrated in many previous experiments with nitrogen addition (Chen et al., 2014; De Beenhouwer et al., 2015; Li et al., 2015). When nutrients were limited, host plants would rely more on AM fungi to obtain nutrients such as soil nitrogen and phosphorus. Nitrogen addition had no significant effect on NRI or NTI of AM fungal communities, and AM fungal species always remained clustered in roots (Figures 6A,C). AM fungal communities that colonized roots were often clustered in natural ecosystems (Saks et al., 2014; Shi et al., 2014). NRI and NTI had a downward trend with increasing nitrogen addition in soil (Figures 6B,D). This also corroborated the finding that Glomeraceae occupied the majority of the AM fungal composition within the roots, but the relative abundance of Glomeraceae in the soil gradually decreased with increasing N fertilization, and new species emerged (Figure 3B). AM fungi occupied different ecological niches in time and space. Some AM fungi existed only in certain soil nutrient conditions.

## Conclusion

In the present study, we found that nitrogen addition reduced the diversity of plant and AM fungal communities in soil by lowering pH and increasing inorganic nitrogen concentration. We suggested that mowing mitigated light limitation by changing the height of plants to alleviate the negative effects of nitrogen addition on the diversity of plant communities, but it could not alleviate the negative effects on AM fungal community. Mowing had important practical significance in grassland management. Whether in soil or roots, AM fungal communities clustered phylogenetically in all treatments, indicating that environmental filtering is the major driving force for AM fungal community assembly. Therefore, our research in future needs to pay more attention to long-term monitoring to explore the feedback between plant and AM fungi, and the response mechanism of AM fungal community to nitrogen deposition.

## Data Availability Statement

The datasets presented in this study can be found in online repositories. The names of the repository/repositories and accession number(s) can be found at: https://www.ncbi.nlm.nih.gov/, PRJNA790751.

## Author Contributions

PZ, GY, and RW conceived the research. SQ, GY, LS, YC, and YZ collected the samples. SQ, LS, YZ, JD, and MS performed the lab analyses. SQ, XL, and NW analyzed the data. PZ, GY, and SQ wrote the manuscript. All authors contributed to the article and approved the submitted version.

## Funding

This work was supported by the Special Foundation for National Science and Technology Basic Resources Investigation of China (2019FY202300); Research Foundation of Qingdao Forest Ecosystem; and Fundamental Research Funds of Shandong University.

## Conflict of Interest

The authors declare that the research was conducted in the absence of any commercial or financial relationships that could be construed as a potential conflict of interest.

## Publisher’s Note

All claims expressed in this article are solely those of the authors and do not necessarily represent those of their affiliated organizations, or those of the publisher, the editors and the reviewers. Any product that may be evaluated in this article, or claim that may be made by its manufacturer, is not guaranteed or endorsed by the publisher.

## Footnotes

^1^ http://qiime.org/install/index.html

^2^ http://maarjam.botany.ut.ee
