# Supplementary material for: Mowing Did Not Alleviate the Negative Effect of Nitrogen Addition on the Arbuscular Mycorrhizal Fungal Community in a Temperate Meadow Grassland
Source: Front Plant Sci. 2022 Jun 9;13:917645. doi: 10.3389/fpls.2022.917645 (PMC9228033; doi:10.3389/fpls.2022.917645)
Supplement: Supplementary file 4 [file Data_Sheet_4.docx]

**Supplementary Information for**

**Mowing did not alleviate the negative effect of nitrogen addition on arbuscular mycorrhizal fungal community in a temperate meadow grassland**

***Siqi Qin* *^1, 2, 3^, Guojiao Yang ^1, 4^, Yang Zhang ^1, 2, 3^, Meixia Song ^1, 2, 3^, Lu Sun ^1, 2, 3^, Yangzhe Cui ^1, 2, 3^, Jibin Dong ^1, 2, 3^, Ning Wang ^1, 2, 3^, Xiao Liu ^1, 2, 3^, Peiming Zheng ^1, 2, 3*^, Renqing Wang ^1, 2, 3^***

*^1^ Institute of Ecology and Biodiversity, School of Life Sciences, Shandong University, 72 Binhai Road, Qingdao 266237, China*

*^2^ Shandong Provincial Engineering and Technology Research Center for Vegetation Ecology, Shandong University, 72 Binhai Road, Qingdao 266237, China*

*^3^ Qingdao Forest Ecology Research Station of National Forestry and Grassland Administration, 72 Binhai Road, Qingdao 266237, China*

*^4^ College of Ecology and Environment, Hainan University, Haikou, 570228, China*

***Correspondence:***Peiming Zheng*
[*zhengpeiming@email.sdu.edu.cn*](mailto:zhengpeiming@email.sdu.edu.cn)

**Supplementary contents:**

**Table. S1** The proportion of variance of AM fungal communities composition explained by the first and second principal components in principal component analysis (PCA).

**Table. S2** Results (F values) of two-way ANOVA for the effects of mowing (M), nitrogen addition (N) and their interactions on soil properties. *P < 0.05, ** P < 0.01, *** P < 0.001.

**Table. S3** Results (F values) of two-way ANOVA for the effects of mowing (M), nitrogen addition (N) and their interactions on plant community α diversity. *P < 0.05, ** P < 0.01, *** P < 0.001.

**Table. S4** Results (F values) of two-way ANOVA for the effects of mowing (M), nitrogen addition (N) and their interactions on α diversity of AM fungal community in roots and soil. *P < 0.05, ** P < 0.01, *** P < 0.001.

**Table. S5** Results (F values) of two-way ANOVA for the effects of mowing (M), nitrogen addition (N) and their interactions on NTI and NRI of the arbuscular mycorrhizal fungal community in roots and soil. *P < 0.05, ** P < 0.01, *** P < 0.001.

**Figure S1** A priori model of the effects of N enrichment and mowing on AM fungal community: (a) AM fungal community in root, (b) AM fungal community in soil.

**Figure S2** Linear regressions of the α-diversity of plant community versus soil pH (a) and inorganic N (b) in unmown treatments.

**Figure S3** Linear regressions of the α-diversity of plant community versus soil pH (a) and inorganic N (b) in mown treatments.

**Figure S4** Rarefaction curves for observed AM fungal OTUs in soil and roots among the different treatments.

**Table. S1** The proportion of variance of AM fungal communities composition explained by the first and second principal components in principal component analysis (PCA).

|  | PC1 | PC2 |
| --- | --- | --- |
| AM fungi in soil | 39.69% | 10.25% |
| AM fungi in root | 37% | 20.32% |

**Table. S2** Results (F values) of two-way ANOVA for the effects of mowing (M), nitrogen addition (N) and their interactions on soil properties. *P < 0.05, ** P < 0.01, *** P < 0.001.

|  | Soil moisture | pH | Total C | Total N | Total P | Available P | 1/NH+ 4-N | Log_10_(NO- 3-N) | 1/Inorganic N |
| --- | --- | --- | --- | --- | --- | --- | --- | --- | --- |
| M | 12.978*** | 0.198 | 0.006 | 0.247 | 0.014 | 0.140 | 0.311 | 0.140 | 0.058 |
| N | 0.780 | 63.447*** | 0.579 | 10.196*** | 1.647 | 1.849 | 15.571*** | 5.922*** | 11.530*** |
| M×N | 0.763 | 2.797* | 0.395 | 0.774 | 1.300 | 0.842 | 1.004 | 1.181 | 1.391 |

**Table. S3** Results (F values) of two-way ANOVA for the effects of mowing (M), nitrogen addition (N) and their interactions on plant community α diversity. *P < 0.05, ** P < 0.01, *** P < 0.001.

|  | Richness | Shannon-Wiener index | Simpson index | Pielou index |
| --- | --- | --- | --- | --- |
| M | 30.534*** | 35.643*** | 40.180*** | 37.421*** |
| N | 11.152*** | 13.823*** | 13.028*** | 14.077*** |
| M×N | 1.777 | 0.173 | 0.243 | 0.159 |

**Table. S4** Results (F values) of two-way ANOVA for the effects of mowing (M), nitrogen addition (N) and their interactions on α diversity of AM fungal community in roots and soil. *P < 0.05, ** P < 0.01, *** P < 0.001.

|  | OUT richness | | Shannon-Wiener index | | Simpson index | | Pielou index | |
| --- | --- | --- | --- | --- | --- | --- | --- | --- |
|  | Root | Soil | Root | Soil | Root | Soil | Root | Soil |
| M | 2.308 | 0.085 | 0.030 | 0.206 | 0.327 | 0.941 | 0.493 | 0.106 |
| N | 2.095* | 1.038 | 3.128* | 2.465 | 2.215 | 4.722** | 1.990 | 3.720* |
| M×N | 1.253 | 0.771 | 0.542 | 0.451 | 0.807 | 0.072 | 1.226 | 0.410 |

**Table. S5** Results (F values) of two-way ANOVA for the effects of mowing (M), nitrogen addition (N) and their interactions on NTI and NRI of the arbuscular mycorrhizal fungal community in roots and soil. *P < 0.05, ** P < 0.01, *** P < 0.001.

|  | Root | | Soil | |
| --- | --- | --- | --- | --- |
|  | NTI | NRI | NTI | Log_10_(NRI) |
| M | 0.712 | 1.031 | 1.836 | 0.490 |
| N | 0.628 | 1.193 | 4.807** | 5.744*** |
| M×N | 0.954 | 1.384 | 0.302 | 0.214 |


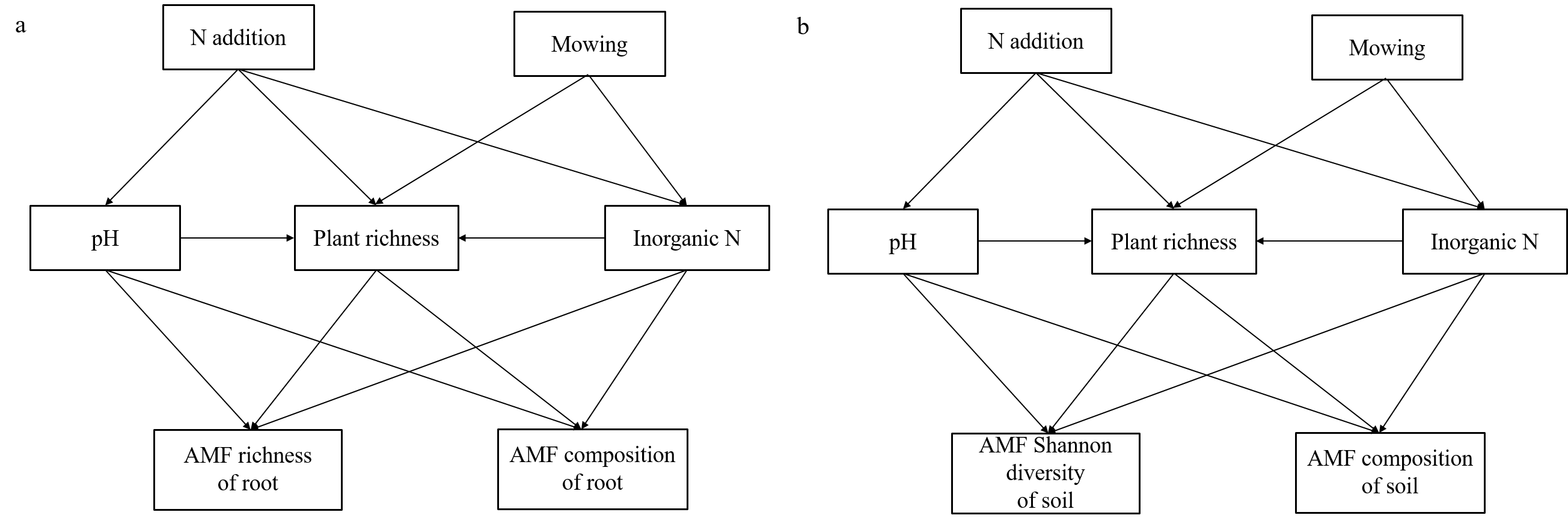


**Figure S1** A priori model of the effects of N enrichment and mowing on AM fungal community: (a) AM fungal community in root, (b) AM fungal community in soil.


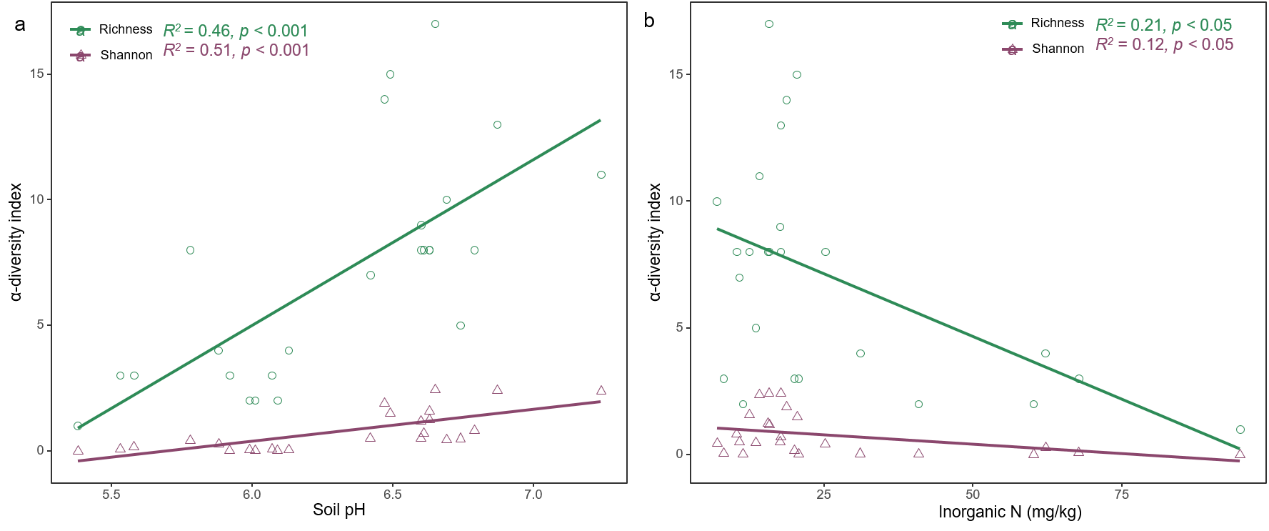


**Figure S2** Linear regressions of the α-diversity of plant community versus soil pH (a) and inorganic N (b) in unmown treatments.

**
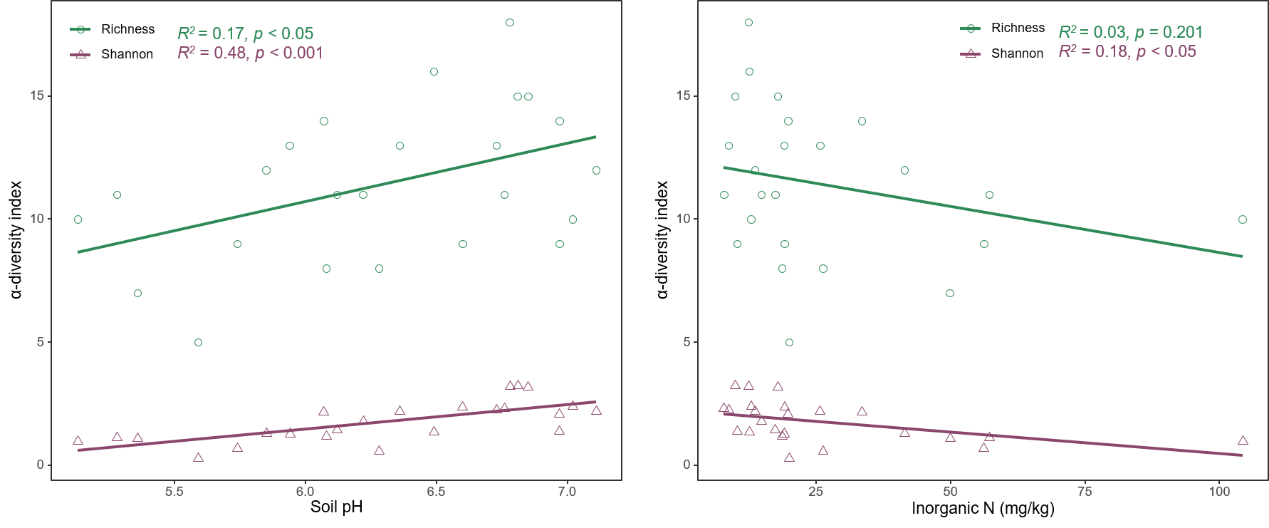
**

**Figure S3** Linear regressions of the α-diversity of plant community versus soil pH (a) and inorganic N (b) in mown treatments.


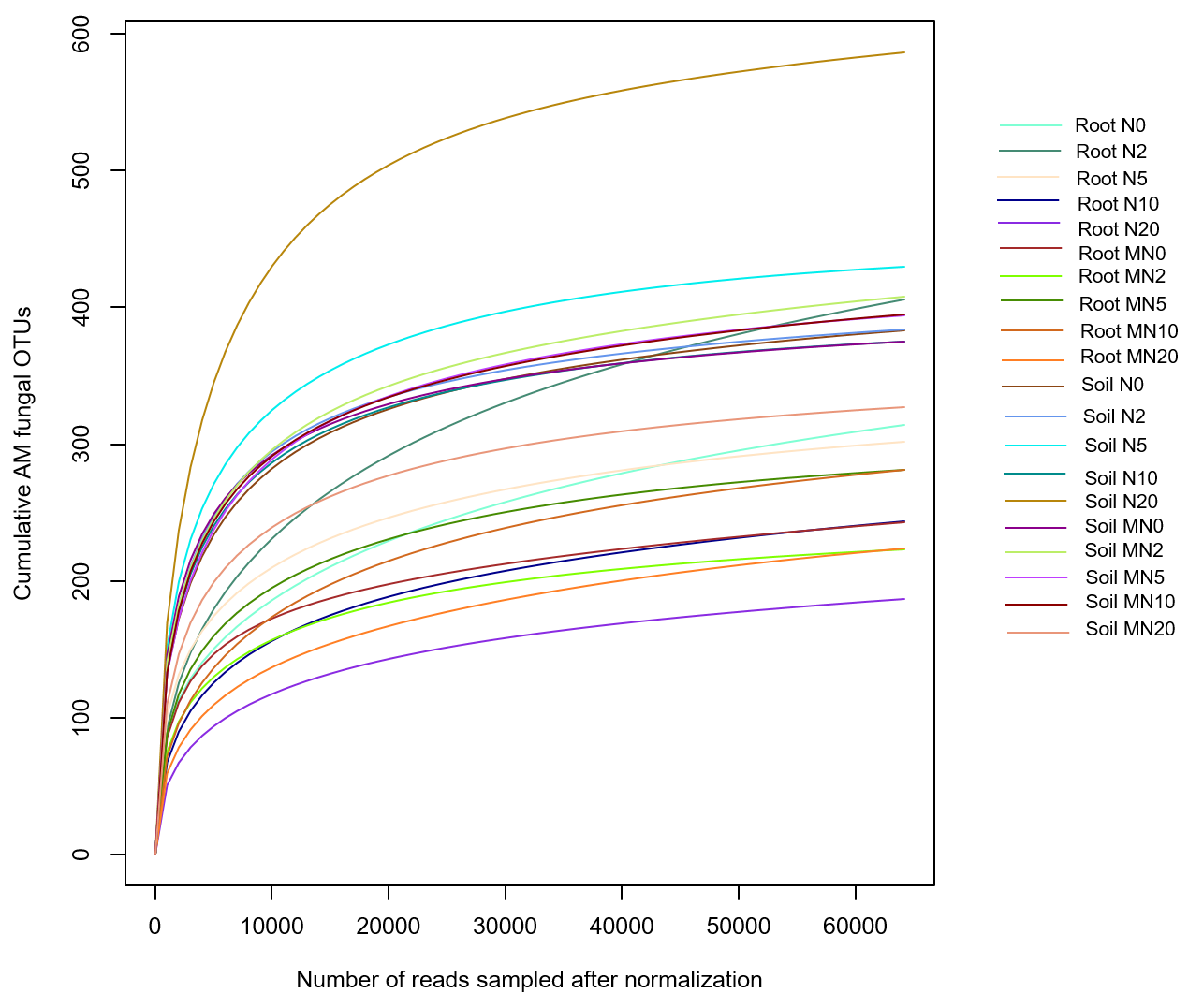


**Figure S4** Rarefaction curves for observed AM fungal OTUs in soil and roots among the different treatments.
